# Disruption of Spike Priming in Virus Entry: Tetrandrine as a Pan‐Coronavirus Inhibitor

**DOI:** 10.1002/mco2.70353

**Published:** 2025-08-31

**Authors:** Kun Wang, Huiqiang Wang, Shuo Wu, Ge Yang, Haiyan Yan, Lijun Qiao, Xingqiong Li, Mengyuan Wu, Jiandong Jiang, Yuhuan Li

**Affiliations:** ^1^ CAMS Key Laboratory of Antiviral Drug Research Beijing Key Laboratory of Technology and Application for Anti‐Infective New Drugs Research and Development NHC Key Laboratory of Biotechnology of Antibiotics Institute of Medicinal Biotechnology Chinese Academy of Medical Sciences and Peking Union Medical College Beijing China; ^2^ State Key Laboratory of Bioactive Substances and Functions of Natural Medicines Institute of Medicinal Biotechnology Peking Union Medical College and Chinese Academy of Medical Sciences Beijing China

**Keywords:** coronavirus, entry, Spike, tetrandrine, transmembrane serine protease 2

## Abstract

The emergence of novel and highly transmissible coronavirus (CoVs) highlights the urgent need for broad‐spectrum antiviral agents. In our pursuit of effective treatments for coronavirus, we identified tetrandrine, the traditional Chinese medicine, as a pan‐coronavirus inhibitor, exhibiting efficacy against HCoV‐229E, HCoV‐OC43, SARS‐CoV‐2, and its major variants of concern (VOCs), including alpha, beta, and omicron. Mechanistic investigations revealed that tetrandrine primarily targets the viral entry stage by binding to the Spike protein, disrupting its interaction with the host protease transmembrane serine protease 2 (TMPRSS2), and promoting Spike protein degradation, ultimately blocking the membrane fusion. Drug resistance selection study identified two mutations, G688R and D814Y, at S2 subunit of Spike, which reduced HCoV‐229E's sensitivity to tetrandrine, supporting its direct action on the viral fusion machinery. Molecular docking and molecular dynamic (MD) simulation together with co‐IP assay also verified the disruption of Spike‐TMPRSS2 complex formation by tetrandrine. Importantly, tetrandrine treatment reduced viral load and mitigated neuropathological damage in infected neonatal mice. These findings establish tetrandrine as a broad‐spectrum coronavirus entry inhibitor and offer mechanistic insights into its antiviral activity, providing a promising candidate for therapeutic development against current and future coronavirus threats.

## Graphical abstract

Tetrandrine inhibits the infection of HCoV‐OC43, HCoV‐229E, SARS‐CoV‐2, and its major variants by blocking virus entry. Specifically, tetrandrine breaks the interaction between TMPRSS2 and Spike, therefore suppress its priming and the following membrane fusion, and induces Spike to degradation.

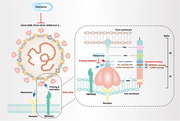

## 1 Introduction

Coronaviruses are enveloped, single‐stranded RNA viruses classified within the order *Nidovirales* and the family *Coronaviridae* [1]. During the evolutionary process, seven coronaviruses have been identified as capable of infecting humans. Among these, four—HCoV‐229E, HCoV‐OC43, HCoV‐NL63, and HCoV‐HKU1—are typically associated with mild respiratory illnesses, predominantly affecting infants and the elderly individuals [2]. However, three highly pathogenic coronaviruses, namely, severe acute respiratory syndrome coronavirus (SARS‐CoV), Middle East respiratory syndrome coronavirus (MERS‐CoV), and recently identified severe acute respiratory syndrome coronavirus 2 (SARS‐CoV‐2), have raised significant global health concerns [3]. Although several targeted antiviral agents against SARS‐CoV‐2—such as Paxlovid (nirmatrelvir/ritonavir) [4, 5], Lagevrio (molnupiravir) [6, 7], Veklury (remdesivir) [8], and azvudine (FNC) [9]—have been developed and authorized for use, the pursuit of new therapeutic strategies remains an urgent priority. This is especially critical for populations at elevated risk, including older adults and individuals with pre‐existing medical conditions, who are more susceptible to severe outcomes.

Viral entry into host cells is a crucial phase in the coronavirus lifecycle and serves as an attractive target for therapeutic intervention. Taking SARS‐CoV‐2 as an example, its entry process is a multistep mechanism involving the Spike (S) protein, a key viral component that facilitates host cell recognition and membrane fusion. This glycoprotein is activated by host proteases such as furin and TMPRSS2, which cleave the Spike protein at the S1/S2 junction following its recognition with the cellular receptor angiotensin‐converting enzyme 2 (ACE2). This cleavage yields two distinct subunits: S1, which harbors the receptor‐binding domain (RBD) responsible for ACE2 interaction, and S2, which remains membrane‐bound and adopts a prefusion conformation. After receptor engagement, structural rearrangements in the S2 subunit lead to the formation of a six‐helix bundle (6‐HB) composed of heptad repeat 1 (HR1) and heptad repeat 2 (HR2), facilitating the fusion of viral and host membranes that enables the viral RNA genome to be released into the host cytoplasm, thereby initiating infection [10, 11]. Considering the pivotal role of this entry step, targeting virus‐ or host‐related components involved in this process offers a promising approach for the development of coronavirus disease 2019 (COVID‐19) therapeutics.

A number of repurposed pharmaceuticals have exhibited in vitro efficacy against SARS‐CoV‐2 by disrupting the virus's ability to enter host cells. Examples include chloroquine [12] and hydroxychloroquine [13], which target the endosomal pathway, and protease inhibitors such as camostat [14] and nafamostat [15] that block the host proteases like TMPRSS2. Additionally, arbidol [16] has demonstrated activity against the viral Spike protein. However, clinical studies have revealed limitations and challenges with these repurposed agents, preventing their widespread adoption as specific antiviral therapies for SARS‐CoV‐2. These outcomes emphasize the urgent need to identify and develop compounds capable of inhibiting viral entry mechanisms.

Traditional Chinese herbal medicines have long contributed to the prevention and treatment of viral infections across China and other parts of Asia. Due to their structural diversity, low toxicity, and broad‐spectrum bioactivities, these natural compounds offer valuable scaffolds for the discovery and development of new antiviral agents. Notably, plant‐derived compounds such as berbamine [17, 18], lycorine [19], and glycyrrhizin [20] have shown antiviral potential against a variety of viruses, including members of the coronavirus family. Among them, tetrandrine, a bizbenzylisoquinoline alkaloid isolated from the perennial climbing herb *Stephania tetrandra S*. Moore [21] (traditionally known as Fang Chi), has emerged as a notable candidate. Tetrandrine has demonstrated antiviral activity against multiple viruses, including herpes simplex virus 1 (HSV‐1) [22], dengue virus [23], Ebola virus [24], MERS‐CoV [25], and SARS‐CoV‐2 [26]. Its antiviral properties are complemented by its anti‐inflammatory, immune‐suppressive, oncological, and cardiovascular effects [21]. As a calcium influx blocker that targets voltage‐gate calcium channels [27], tetrandrine is also approved in China for managing silicosis.

In this study, using an array of in vitro and in vivo models, we provide evidence that tetrandrine exerts strong antiviral effects against HCoV‐229E, HCoV‐OC43, SARS‐CoV‐2, and its variants. Mechanistically, this activity appears to result from interference with viral entry, particularly through modulation of the Spike protein. Tetrandrine promotes Spike protein degradation and disrupts its interaction with the host protease TMPRSS2. These findings position tetrandrine as a promising candidate for the continued development of coronavirus entry inhibitors.

## 2 Results

### 2.1 Identification of Tetrandrine as a Potent Anti‐Common Coronavirus Compound

The chemical structure of tetrandrine is shown in Figure S1A; in advance of evaluating the antiviral activity, we first tested the cytotoxicity of tetrandrine on multiple cell lines. Note that 50% cytotoxic concentration (CC_50_) of tetrandrine on tested cell lines was calculated and shown in Figure S1B–F. We chose non‐toxic concentrations of tetrandrine to exclude the antiviral activity caused by cytotoxicity in the future studies.

**FIGURE 1:**
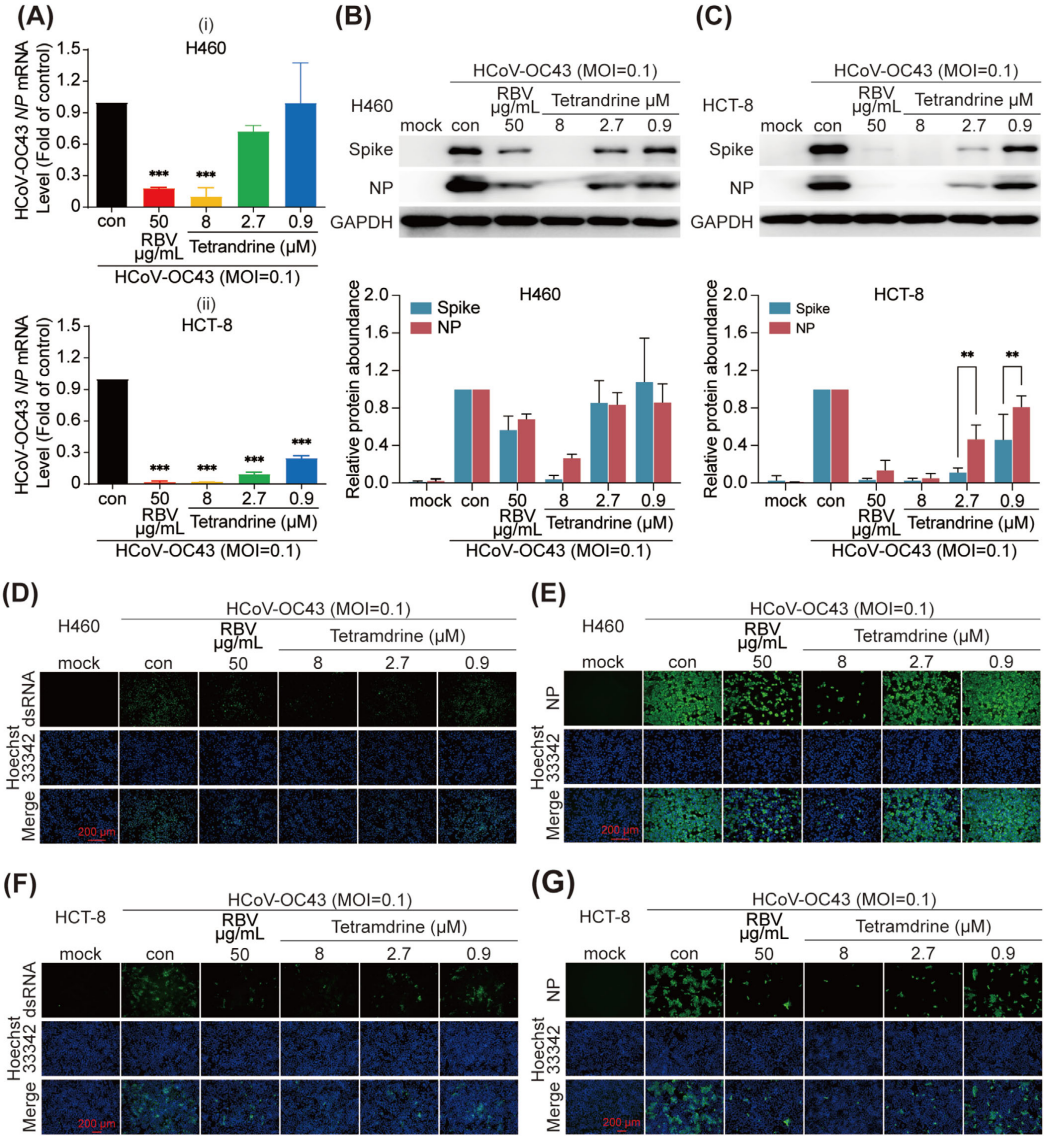
Tetrandrine suppressed HCoV‐OC43 replication in various cell lines. H460 and HCT‐8 cells were infected with HCoV‐OC43 and concurrently treated with indicated concentrations of tetrandrine for 24 h. (A) Viral RNA levels were quantified using qRT‐PCR assay. Statistical significance was evaluated by one‐way ANOVA (mean ± SD, *n* = 3). *p < 0.05, **p < 0.01, ***p < 0.001 compared to the virus control group. (B and C) Expression of viral proteins was assessed by immunoblotting. Each experiment was independently repeated three times, and statistical analysis was conducted using two‐way ANOVA (mean ± SD, *n* = 3). *p < 0.05, **p < 0.01, ***p < 0.001 versus virus control. For panels (D–G), the levels of viral double‐stranded RNA (dsRNA) (D, F) and the nucleocapsid protein (NP) (E, G) were visualized via immunofluorescence staining. Scale bar = 200 µm. Con, HCoV‐OC43‐infected group; RBV, ribavirin‐treated group.

**FIGURE 2:**
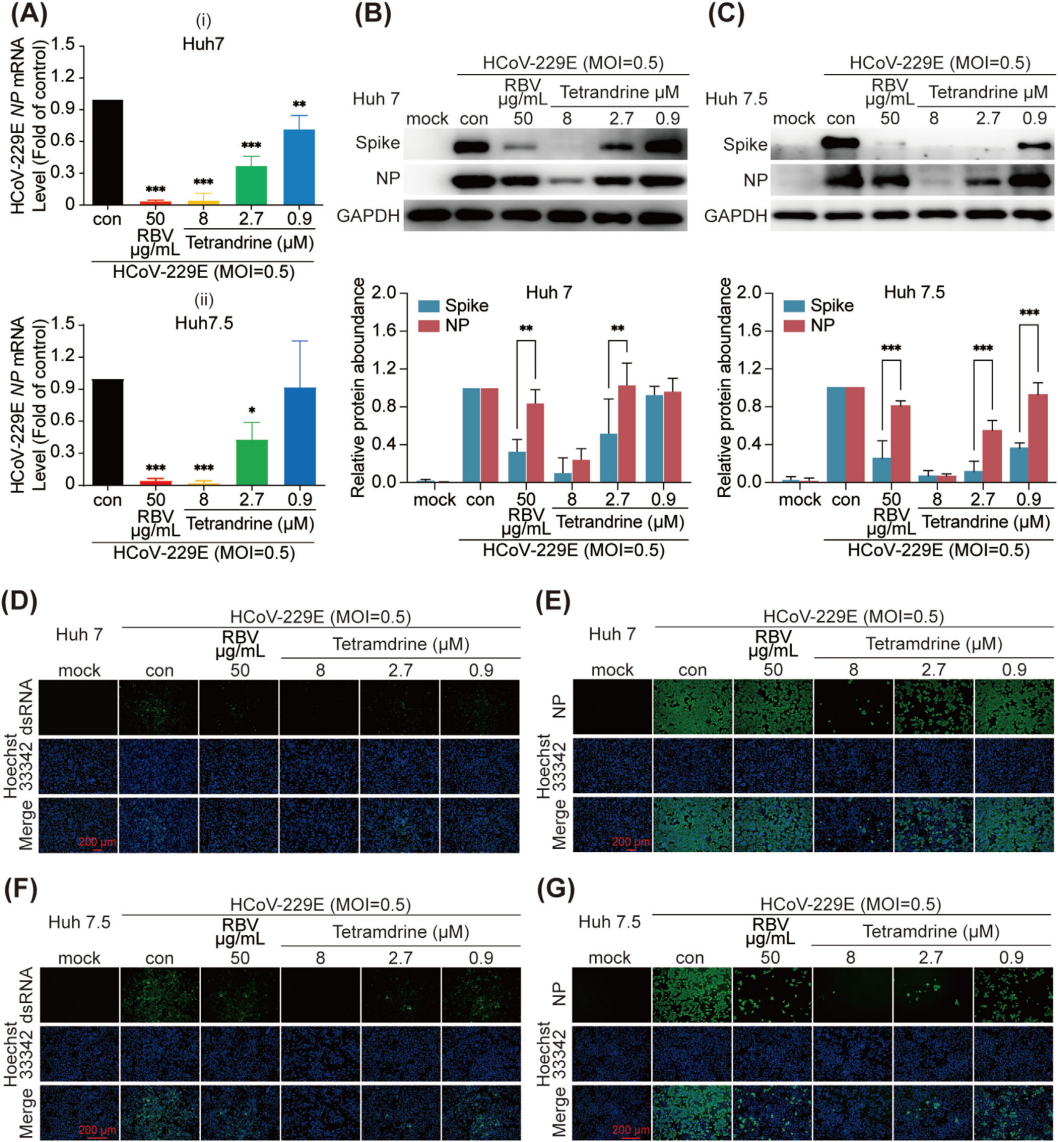
Tetrandrine suppressed HCoV‐229E replication in various cell lines. Huh 7 and Huh 7.5 cells were infected with HCoV‐229E and concurrently treated with indicated concentrations of tetrandrine for 24 h. (A) Viral RNA levels were quantified using qRT‐PCR assay. Statistical significance was evaluated by one‐way ANOVA (mean ± SD, *n* = 3). *p < 0.05, **p < 0.01, ***p < 0.001 compared to the virus control group. (B and C) Expression of viral proteins was assessed by immunoblotting. Each experiment was independently repeated three times, and statistical analysis was conducted using two‐way ANOVA (mean ± SD, *n* = 3). *p < 0.05, **p < 0.01, ***p < 0.001 versus virus control. For panels (D–G), the levels of viral double‐stranded RNA (dsRNA) (D, F) and the nucleocapsid protein (NP) (E, G) were visualized via immunofluorescence staining. Scale bar = 200 µm. Con, HCoV‐229E‐infected group; RBV, ribavirin‐treated group.

HCoV‐OC43‐infected H460 or HCT‐8 cells and HCoV‐229E‐infected Huh 7 or Huh 7.5 cells were used to examine the antiviral effect of tetrandrine. As shown in Figures 1A and 2A, tetrandrine exhibited a dose‐dependent inhibition of RNA levels for both HCoV‐229E and HCoV‐OC43 in the designated cell lines. Meanwhile, double‐stranded RNA (dsRNA), an intermediate product of virus replication process, visualized a similar dose‐dependent inhibitory effect in both HCoV‐229E‐ and HCoV‐OC43‐infected cells treated with tetrandrine (Figures 1D,F and 2D,F).

To evaluate the antiviral activity at the protein level, we selected two essential structural components: nucleocapsid (N) protein and Spike (S) protein. Our results demonstrated that tetrandrine suppressed the expression of both NP and Spike proteins from HCoV‐OC43 and HCoV‐229E in a dose‐dependent manner, as evidenced by immunoblotting (Figures 1B,C and 2B,C) and immunofluorescence (Figures 1E,G and 2E,G) assays. Notably, the Spike protein exhibited greater sensitivity to lower doses of tetrandrine than NP, with this effect being more pronounced in HCoV‐229E‐infected cells (Figures 1B,C and 2B,C). The NP primarily associates with viral RNA to form a protective complex that facilitates genome packaging and participates in viral transcription and replication processes. In contrast, the Spike glycoprotein mediates viral attachment to host receptors and promotes membrane fusion, enabling the virus to enter target cells [10].

In summary, these findings indicate that tetrandrine treatment significantly inhibits the expression of HCoV‐OC43 and HCoV‐229E proteins, as well as intracellular viral RNA and dsRNA, across all tested cell lines.

### 2.2 Tetrandrine Inhibited SARS‐CoV‐2 and Its Variants

**FIGURE 3:**
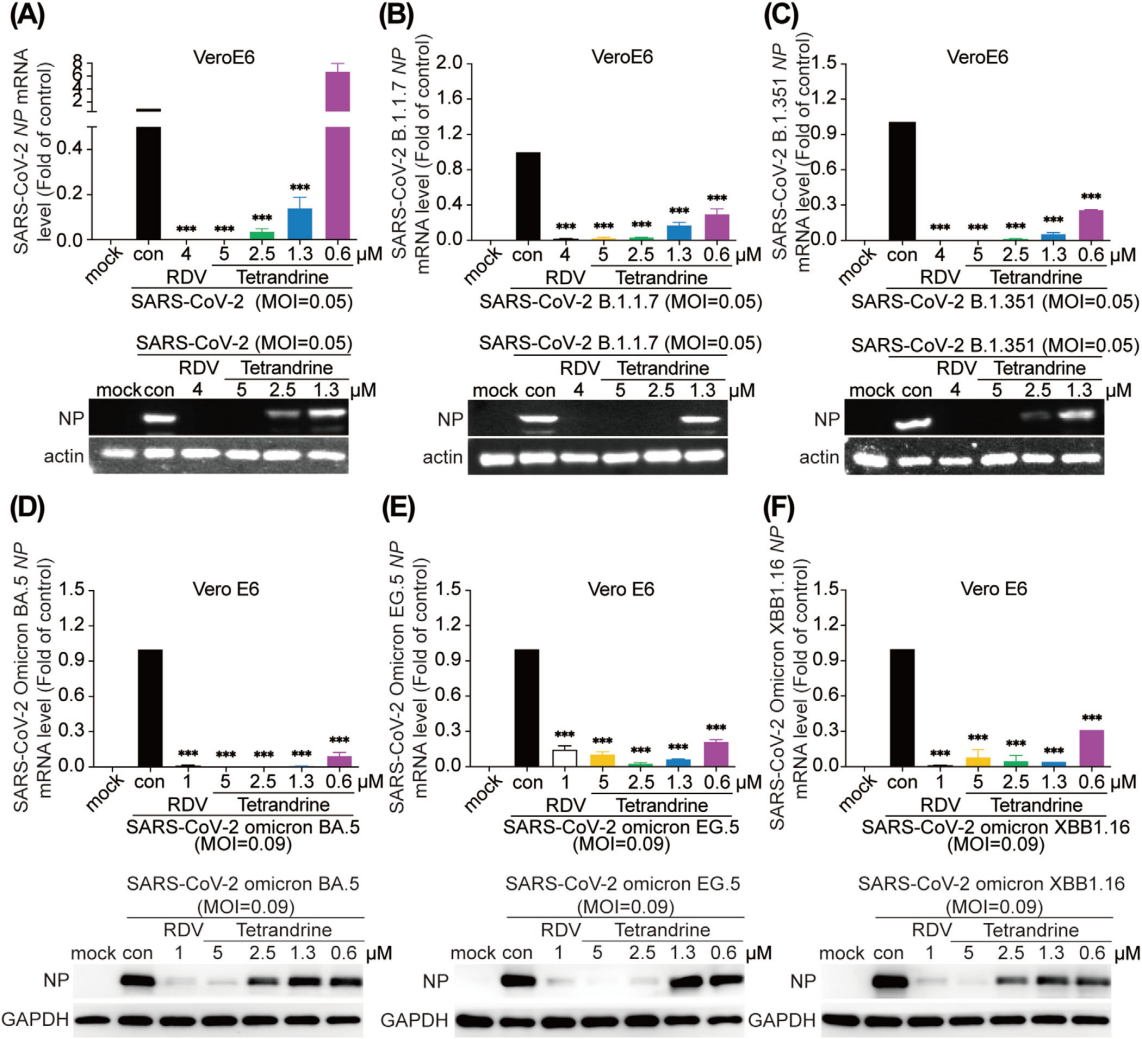
Tetrandrine suppressed the replication of SARS‐CoV‐2 and its variants in Vero E6 cells. Vero E6 cells were infected with SARS‐CoV‐2 or variants (B.1.1.7, B.1.351, omicron BA.5, EG.5, and XBB1.16), followed by treatment with various concentrations of tetrandrine for 24 h. Viral replication was assessed by quantifying NP RNA and protein levels using qRT‐PCR and immunoblotting assays, respectively (A–F). Statistical analysis was performed using one‐way ANOVA (mean ± SD, *n* = 3). *p < 0.05, **p < 0.01, ***p < 0.001 compared to virus control. Mock, cell group; Con, SARS‐CoV‐2‐infected group. RDV, remdesivir‐treated group.

As tetrandrine showed impressive pharmacological activity on common coronaviruses, we then investigated whether SARS‐CoV‐2 and its variants could be inhibited as well. Here, SARS‐CoV‐2, along with the alpha (B.1.1.7) variant first monitored in the UK, the beta (B.1.351) variant identified in South Africa, and three lately occurred omicron variants (BA.5, EG.5, and XBB1.16) were infected with Vero E6 cells and treated with tetrandrine simultaneously. The NP of all six strains was dose‐dependently inhibited at both mRNA and protein levels (Figure 3), consistent with the previous reports [28, 29]. Tetrandrine exhibited a more potent inhibitory effect on the five SARS‐CoV‐2 variants compared to the original strain (Figure 3B–F). Taken together, the aforementioned findings highlight tetrandrine's broad‐spectrum inhibitory effects on coronaviruses, including common coronaviruses, the newly emerged SARS‐CoV‐2, and its variants.

### 2.3 Tetrandrine Targeted on the Entry Process of Coronavirus

**FIGURE 4:**
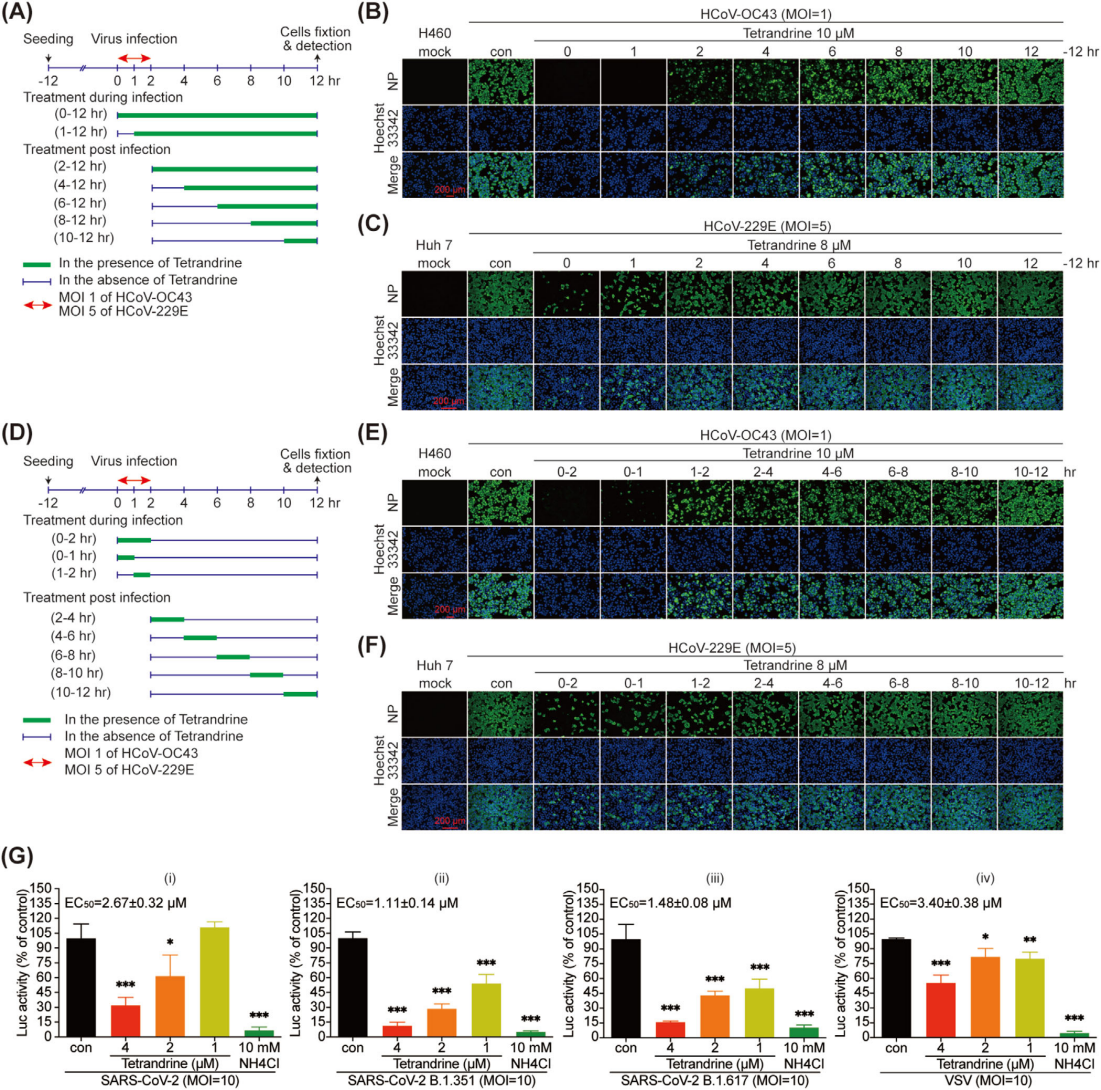
Tetrandrine efficiently inhibited the infection of CoVs by blocking entry events. (A–F) H460 and Huh 7 cells were infected with HCoV‐OC43 and HCoV‐229E, respectively. The experimental procedures of time‐of‐addition assay (A) and time course assay (D) are illustrated, with tetrandrine administered at specified concentrations at different time points (B, C) or during defined time intervals (E, F). At 12 h post‐infection, cells were fixed, and viral NP protein levels were detected using immunofluorescence. Scale bar: 200 µm. (G) HEK293T‐hACE2 cells were exposed to pseudotyped SARS‐COV‐2 (wild type and two variants: B.1.351 and B.1.671) or VSV (MOI = 10) in the presence of varying concentrations of tetrandrine or NH_4_Cl. Firefly luciferase activity, indicative of infection, was measured 24 h post‐infection and normalized to the mock‐treated control. Statistical analysis was performed using one‐way ANOVA (mean ± SD, *n* = 3). *p < 0.05, **p < 0.01, ***p < 0.001 versus virus control. Mock, cell group. Con, virus‐infected group.

Next, to investigate the specific process of coronavirus lifecycle targeted by tetrandrine, a time‐of addition assay (Figure 4A) was performed. It was observed that in both HCoV‐OC43‐ and HCoV‐229E‐infected cells, the antiviral effect of tetrandrine also diminished with delayed addition time. Notably, tetrandrine added at 0–6 h post‐infection still showed an anti‐HCoV‐OC43 effect to different degree, while the anti‐viral effect on HCoV‐229E only manifested at 0–1 h post‐infection (Figure 4B,C), indicating that tetrandrine could target the early stage of CoVs lifecycle. Meanwhile, a time‐course assay (tetrandrine treatment 1 or 2 h) was also conducted as described (Figure 4D) to further specify the above results. The anti‐HCoV‐OC43 effect of tetrandrine was specified to 0–2, 0–1, 1–2, 2–4, and 4–6 h post‐infection, while the anti‐HCoV‐229E effect was focused on 0–2, 0–1, and 1–2 h post‐infection (Figure 4E,F). The above results indicated the effective treatment windows of 0–2, 0–1, and 1–2 h, which emphasized the significance of tetrandrine in the entry process of coronaviruses lifecycle.

Furthermore, a pseudovirus experiment was performed to assess tetrandrine's potential inhibition of the entry process. The results, shown in Figure 4G, suggested that tetrandrine could suppress the entry of both SARS‐CoV‐2 pseudovirus and the variants (B.1.351 and B.1.617) pseudoviruses, which were reported to harbor 10 and 12 mutations in Spike gene compared to the original strain, respectively [30], in previous studies. The EC_50_ of tetrandrine on the entry process of the two variants were lower than the wild‐type SARS‐CoV‐2, in agreement with the better inhibitory effect to SARS‐CoV‐2 variants showed before (Figure 3). Additionally, tetrandrine also showed an inhibitory activity against VSV pseudovirus entry, with a lesser extent than it to SARS‐CoV‐2. Therefore, the above results suggest tetrandrine against coronaviruses by targeting viral entry.

### 2.4 Tetrandrine Targeted on Spike and Induce It to Degradation

**FIGURE 5:**
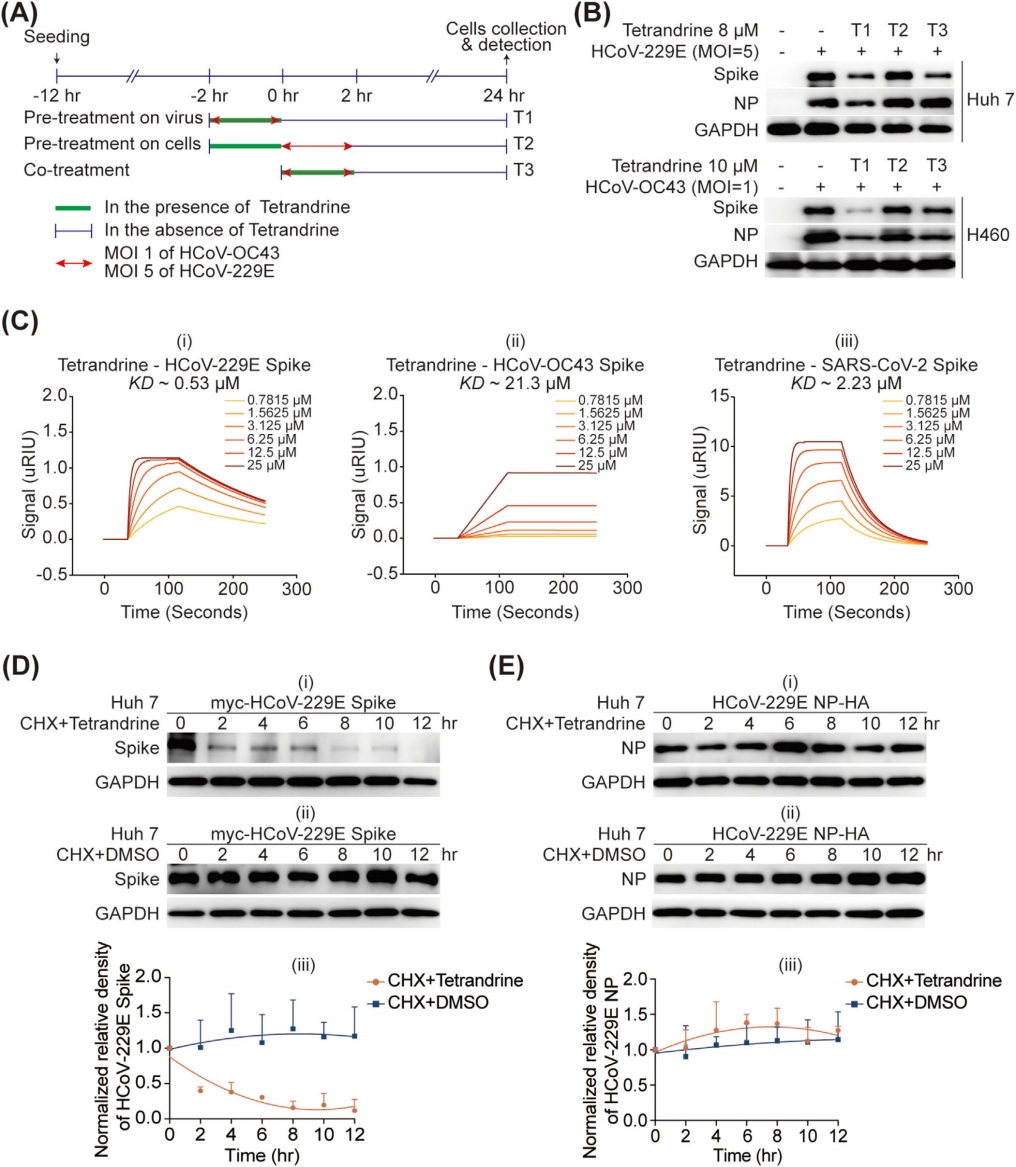
Tetrandrine interacted with Spike protein of pan‐coronaviruses and induced HCoV‐229E Spike to degradation. (A) Evaluation of the effective treatment window for tetrandrine. (B) Huh 7 and H460 cells were infected with HCoV‐229E or HCoV‐OC43 and treated with tetrandrine at various stages—prior to infection, during viral entry or post infection. At 24 h post infection, cells were collected, and viral protein expression was assessed by immunoblotting analysis. (C) Surface plasmon resonance (SPR) assays were conducted using purified Spike proteins from HCoV‐229E, HCoV‐OC43, and SARS‐CoV‐2, which were immobilized on CM5 sensor chips. Gradient concentrations of tetrandrine were sequentially injected, and real‐time binding responses were recorded to evaluate the interaction between tetrandrine and each viral Spike protein. (D–E) Huh 7 cells were transfected with 1 µg of pLV‐myc‐HCoV‐229E Spike or pCMV3‐HCoV‐229E NP‐HA plasmids and incubated for 24 h. Cycloheximide (20 µg/mL) was administered concurrently with either 5 µM tetrandrine or vehicle (DMSO). Cells were harvested at designated time points following treatment, and the levels of Spike (D) and NP (F) proteins were analyzed via immunoblotting. All experiments were conducted in triplicate.

To investigate whether tetrandrine participates in the entry phase by targeting the virus, we pre‐incubated tetrandrine either with viruses or cells for 2 h before infection, with tetrandrine treated simultaneously with infection serving as the control (Figure 5A). As shown in Figure 5B, tetrandrine pre‐incubated (−2 to 0 h) with cells exhibited no antiviral effects, while tetrandrine pre‐incubated (−2 to 0 h) with viruses showed same antiviral effects as co‐treatment did in both HCoV‐229E‐ and HCoV‐OC43‐infected cells, with a more pronounced inhibitory effect on Spike protein compared to NP, consistent with Figures 1B,C and 2B,C. These results imply that tetrandrine functions during the entry phase and may target the viral Spike protein. Therefore, we further designed surface plasmon resonance (SPR) experiment to observe whether tetrandrine could directly operate with Spike. Through gradient diluting tetrandrine, we observed its interaction with the Spike proteins of various coronaviruses in vitro (Figure 5C). A more robust binding capacity was identified between tetrandrine and HCoV‐229E's Spike compared to that with SARS‐CoV‐2 and HCoV‐OC43. Thus, we selected HCoV‐229E as a representative case to delve further into the impact of tetrandrine on the Spike protein.

Cells transfected with the plasmid expressing myc‐tagged HCoV‐229E Spike were treated with tetrandrine in the presence of cycloheximide (CHX). These results proved that the expression level of Spike was suppressed by tetrandrine, while the solvent DMSO did not alter its expression (Figure 5D). Moreover, HCoV‐229E NP tagged with HA was also subjected to the same experiment to demonstrate that the degradation‐inducing effect of tetrandrine on the Spike protein was specific (Figure 5D,E).

### 2.5 HCoV‐229E Developed Resistance to Tetrandrine by Mutating Spike

**FIGURE 6:**
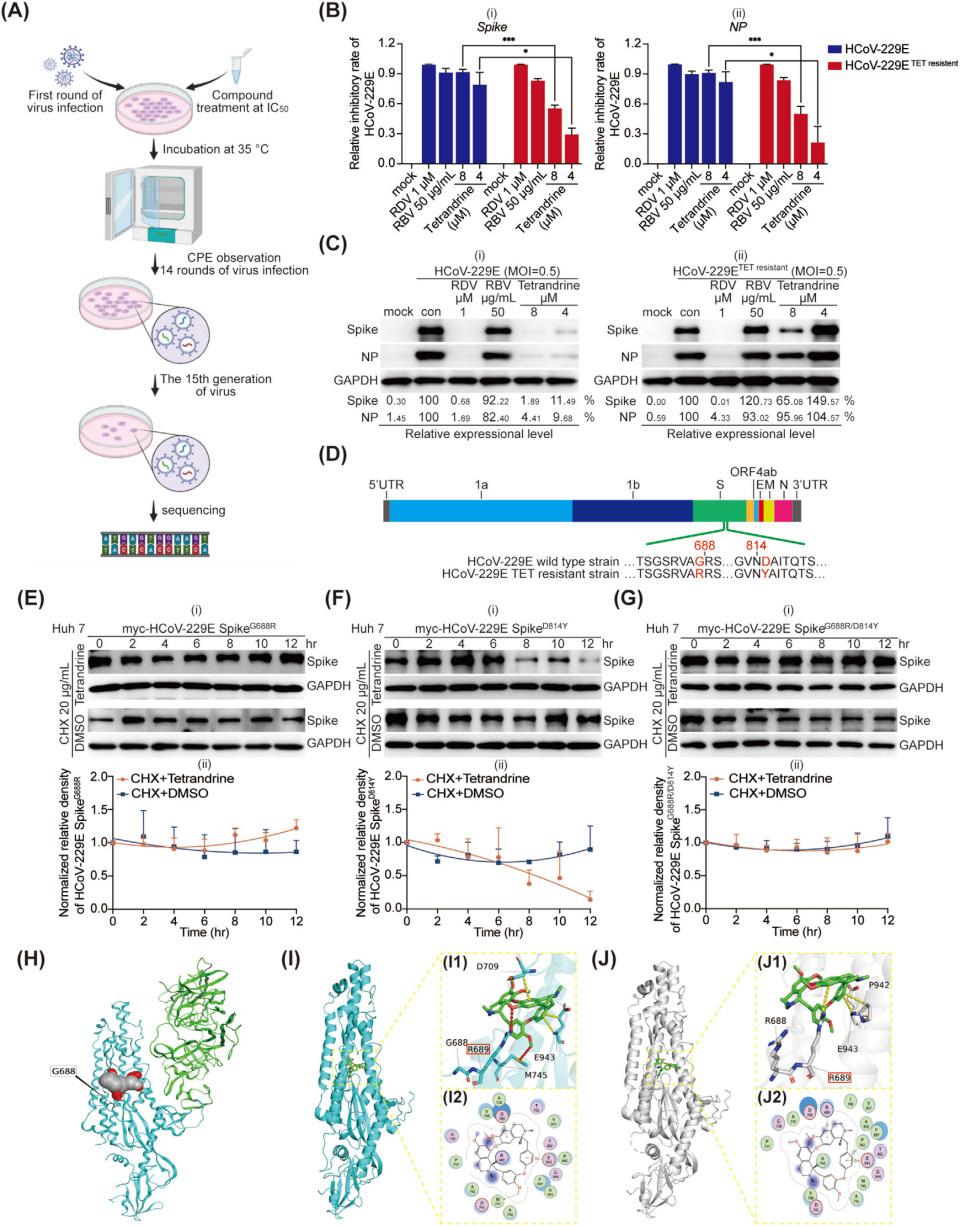
Tetrandrine induced mutation of HCoV‐229E at S2 subunit of Spike gene. (A) Schematic representation of the experimental procedure used to generate tetrandrine‐resistant HCoV‐229E strains (generated with elements form Biodender). (B and C) Huh 7 cells were infected with wild‐type and tetrandrine‐resistant HCoV‐229E, respectively, and treated with indicated concentrations of tetrandrine simultaneous. At 24 h post‐infection, total RNA and protein were extracted for qRT‐PCR (B) and immunoblotting (C) to evaluate viral replication. Each experiment was independently repeated three times. Statistical significance was determined using multiple *t*‐test (mean ± SD, *n* = 3). *p < 0.05, **p < 0.01, ***p < 0.001 versus virus control. Mock, cell control group. Con, virus‐infected group. RBV, ribavirin‐treated group. RDV, remdesivir‐treated group. (D) Huh 7 cells were infected with wild‐type and tetrandrine‐resistant HCoV‐229E, harvested at 24 h post‐infection, and subjected to Trizol‐based RNA extraction. Sequencing analysis (MAGIGENE platform) revealed two point mutations—G688R and D814Y—located within the S2 domain of the Spike gene. (E–G) Huh 7 cells were infected with 1 µg pLV‐myc‐HCoV‐229E Spike^G688R^, pLV‐myc‐HCoV‐229E Spike^D814Y^, or pLV‐myc‐HCoV‐229E Spike^G688R/D814Y^, respectively, and incubated for 24 h. Note that 20 µg/mL cycloheximide was added together with 5 µM tetrandrine or DMSO. At designated time points post‐treatment, cells were lysed, and expression levels of Spike^G688R^ (E) and Spike^D814Y^ (F) and Spike^D814Y/D814Y^ (G) were analyzed by immunoblotting. Quantitative densitometry was conducted using ImageJ, and results from triplicate experiments were presented. (H) Overview of predicted binding between tetrandrine and the Spike S2 domain. (I and J) The overall view of binding conformation of tetrandrine and Spike S2 domain (I) or G688R mutant S2 domain (J), 3D partial view of tetrandrine in Spike S2 domain (I1), or G688R mutant Spike S2 domain (J1); the carbon atom of tetrandrine is green, the oxygen atom is red, the nitrogen atom is blue, and the wide‐type Spike protein is shown as cyan Cartoon, the G688R mutant Spike protein is shown as gray Cartoon. The surrounding amino acids are marked in black font, and the dotted yellow line indicates arene‐H stacking and arene‐cation stacking effects. 2D partial view of tetrandrine in Spike S2 domain (I2) or G688R mutant Spike S2 domain (J2), and green dashed lines indicate arene‐H stacking and arene‐cation stacking effects.

Meanwhile, we selected resistant virus by continuously passaging HCoV‐229E in the presence of tetrandrine, as described in Figure 6A. After 14 generations of passaging, tetrandrine‐resistant HCoV‐229E was obtained. In comparison with wild‐type HCoV‐229E, we found that the tetrandrine‐resistant strain of HCoV‐229E exhibited less sensitivity to tetrandrine, as expected, while remdesivir and ribavirin treatments showed no significant differences (Figure 6B,C). Furthermore, through sequencing and alignment of the tetrandrine‐resistant HCoV‐229E variant and the wild‐type strain, we observed two mutations, G688R and D814Y, in S2 domain of Spike gene (Figure 6D). As was known, the S2 region of the Spike gene in coronaviruses, including SARS‐CoV‐2, was conserved and played a critical role in the process of viral entry. This result was in agreement with our former assumption that tetrandrine was an entry inhibitor and targeted the Spike protein. Hence, we were eager to learn whether the two mutations were related to tetrandrine's inhibition of the Spike protein.

We initially investigated whether the two mutations affected tetrandrine's ability to degrade the Spike protein. Plasmids of myc‐tagged Spike^G688R^, Spike^D814Y^ or Spike^G688R/D814Y^ were transfected into the cells and tetrandrine was added together with CHX. Surprisingly, tetrandrine lost its ability to degrade Spike^G688R^ and Spike^G688R/D814Y^, while Spike^D814Y^ could still be degraded slowly (Figure 6E–G), indicating that the Gly688 might play a critical role during Spike degradation induced by tetrandrine. Through a molecular docking analysis, we observed that tetrandrine was located at the S2 subunit of Spike (Figure 6H), binding with the residues Met745, Arg689, Asp709, and Glu943 instead of Gly688 or Asp814 (Figure 6I). Notably, among all predicted binding sites, Arg689 was located at the fusion peptide and precisely at the conserved S2 cleavage site targeted by host protease TMPRSS2, adjacent to Gly688 we observed. This result reminded us that the binding of tetrandrine to the HCoV‐229E Spike protein might be associated with the priming process facilitated by the host protease. Moreover, the mutation Arg688 led to an elongation of the amino acid side chain compared to Gly688, which might cause an altered interaction mode between tetrandrine and Spike at S2 subunit, and further interfered with the priming and activation of Spike (Figure 6J).

### 2.6 Tetrandrine Targeted and Broke Spike Priming Mediated by TMPRSS2

**FIGURE 7:**
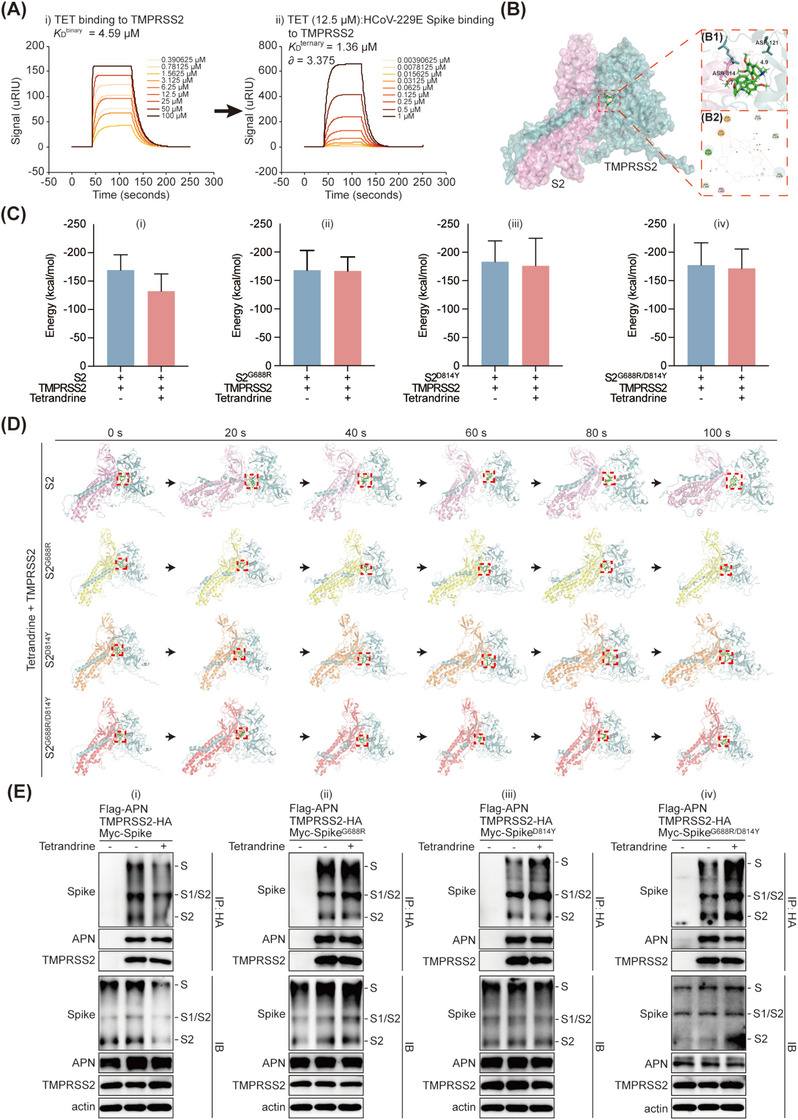
Tetrandrine formed ternary complex with TMPRSS2 and Spike therefore interfered with Spike priming process. (A) To assess the interaction between tetrandrine, Spike, and TMPRSS2, purified hTMPRSS2 protein was immobilized on a CM5 SPR sensor chip. A concentration gradient of tetrandrine (i), or Spike protein pre‐mixed with 12.5 µM tetrandrine (ii), was sequentially injected into the SPR flow channels. Real‐time sensor grams were recorded to evaluate binding interactions, and the cooperativity coefficient (*α* value) was calculated [32] to assess ternary complex formation. (B) Molecular docking simulations were employed to predict the binding interfaces among tetrandrine, the S2 subunit of Spike and TMPRSS2. Both 3D (B1) and 2D (B2) visualizations of predicted binding conformations are presented. Yellow dashed line highlight arene‐H stacking interactions. (C and D) To further validate the stability of the ternary complex, 100‐ns MD simulations were performed involving tetrandrine, TMPRSS2, and the specified S2 domains. The binding free energy (C) was estimated using the molecular mechanics Poisson–Boltzmann surface area (MM‐PBSA) method. Representative simulation snapshots (D) were captured every 20 s to illustrate the structural dynamics of the complex. (E) Huh 7 cells were co‐transfected with the indicated plasmids for 24 h. 8 µM tetrandrine or DMSO were then added into the cells and incubated for another 24 h. The interaction among indicated proteins were determined with immunoprecipitation assay and then analyzed by immunoblotting.

TMPRSS2, type II transmembrane serine protease (TTSP), plays a crucial role in various physiological processes, including the activation of Spike protein when coronaviruses enter the cell by cleavage Spike at specific sites, the activated S2 subunit subsequently facilitates the fusion of viral membrane with the host cell membrane [31]. Through SPR assay, we validated that tetrandrine could also bind to TMPRSS2, and form a ternary complex with TMPRSS2 and S2 subunit, based on the method reported by Roy et al. [32] (Figure 7A), molecular docking also confirmed this result and predicted that tetrandrine simultaneously bind to Asp814 of Spike and Asp121 of TMPRSS2 (Figure 7B), demonstrating its involvement in Spike priming process. As Asp814 was substituted with the mutation Tyr814 in HCoV‐229E tetrandrine resistant strain, which could lead to the change of interaction among the ternary complex, and the activation of Spike no longer affected.

Therefore, we conducted molecular dynamics simulations to access the free energy of the system after tetrandrine bound to the complex. When tetrandrine was added into the system, the free energy between S2 and TMPRSS2 decreased significantly from −169.3± 27.0 kcal/mol to −132 ± 30.53 kcal/mol, demonstrating that tetrandrine disrupted the interaction between S2 and TMPRSS2. However, with S2 subunit mutant, no matter G688R or D814Y or double mutation, tetrandrine had no significant effect on the free energy between Spike and TMPRSS2 (Figure 7C). Meanwhile, the binding dynamics of the ternary complex was captured every 20 ns, we observed that S2‐TMPRSS2–tetrandrine complex became much looser as time prolonged compared to other three S2 mutants formed complexes (Figure 7D), which corroborating the observed destabilization of free energy and once again demonstrated that tetrandrine interfered the activation of S2 primed by TMPRSS2.

Since the priming of Spike requires the involvement of cellular receptors and host proteases, we co‐transfected plasmids expressing myc‐tagged HCoV‐229E Spike and Flag‐tagged membrane receptor APN, along with plasmid expressing HA‐tagged TMPRSS2, into Huh 7 cells to simulate the cleavage process of HCoV‐229E Spike. Based on this in vitro system, we examined the effect of tetrandrine on Spike priming process, including both wild type and the three mutants (S^G688R^, S^D814Y^, and S^G688R/D814Y^). Our results demonstrated that in the presence of APN and TMPRSS2, the co‐transfected Spike or the mutants were all primed, with the detectable S1/S2 and S2 bands. However, when tetrandrine was added, the interaction between wild‐type Spike and TMPRSS2 was impaired, leading to reduced Spike priming, while the interaction between TMPRSS2 and S^G688R^, S^D814Y^ or S^G688R/D814Y^, as well as their priming process were not affected (Figure 7E). These results indicated that tetrandrine interferes with TMPRSS2‐mediated Spike priming.

### 2.7 Tetrandrine Exerts Anti‐HCoV‐OC43 Effect In Vivo

**FIGURE 8:**
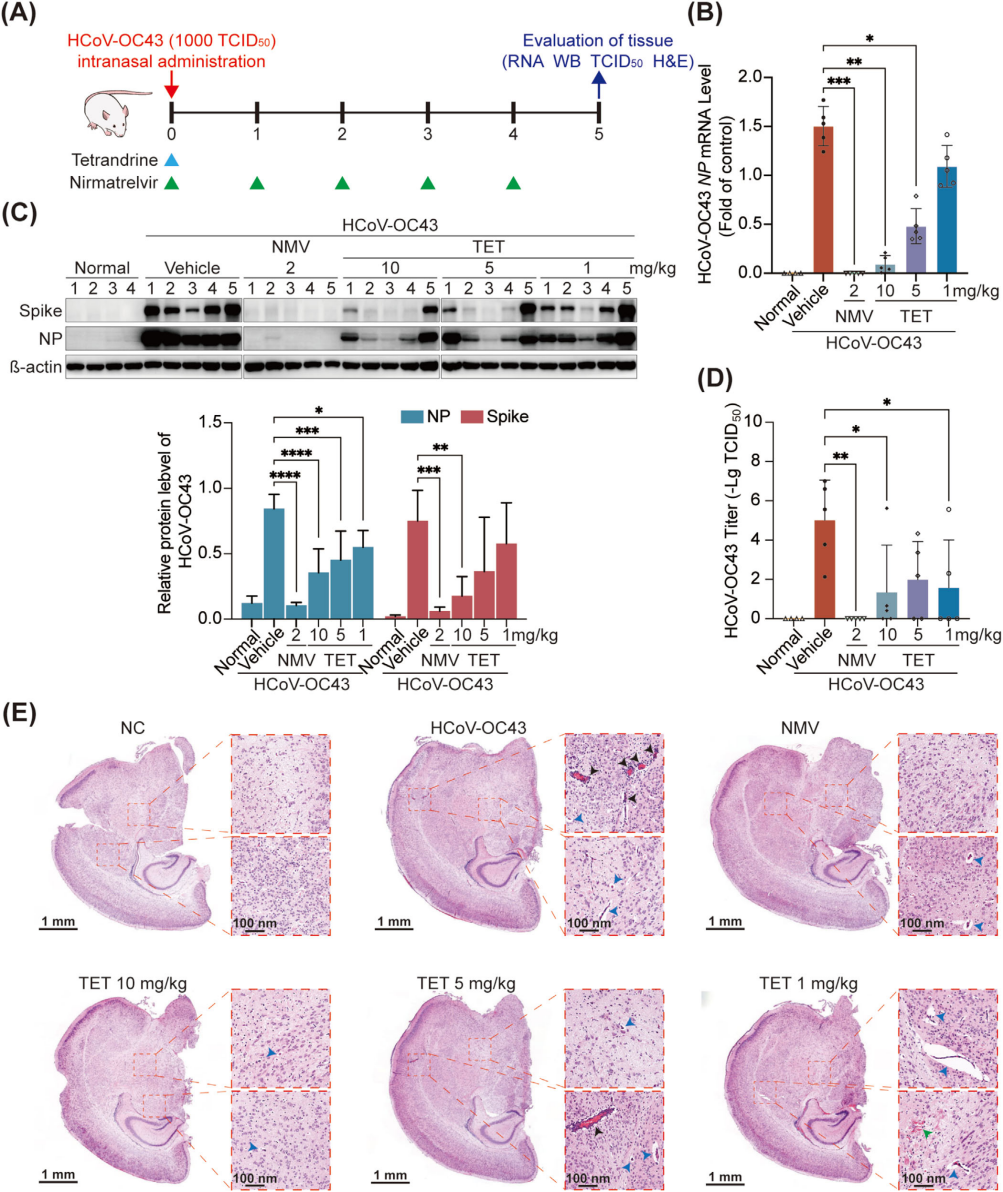
Tetrandrine exerted antiviral efficacy in vivo. (A) Schematic illustration of the experimental protocol used in the animal study (generated with elements form BioArt). (B–D) Viral burden in mouse brain tissues was assessed using three complementary approaches: qRT‐PCR assay with virus‐specific primers (B, *n* = 5), immunoblotting analysis with targeted antibodies (C, *n* = 5), and the viral titration by the end‐point dilution assay (TCID_50_) (D, *n* = 5) in H460 cells. (E) Brain tissues were harvested at five days post‐infection (dpi), fixed, embedded in paraffin, sectioned, and subjected to hematoxylin and eosin (H&E) staining. Histopathological changes were annotated as follows: blue arrow heads, neuron vacuolation (hydropic degeneration); black arrow heads, perivascular cuffing; green arrow heads, small hemorrhagic lesion.

To evaluate whether tetrandrine could protect mice from coronavirus infection through its entry inhibition mechanism, HCoV‐OC43‐infected neonatal mouse model was applied; as shown in Figure 8A, 7‐day‐old Balb/c mice were intranasal inoculated with 10 LD_50_ (1000 TCID_50_) of HCoV‐OC43. Tetrandrine (10, 5, and 1 mg/kg) was administered once right after the viral infection to mice via intranasal instillation. A solvent‐administered post‐infection served as the vehicle control. Nirmatrelvir, given orally once per day, functioned as the positive control. At 5 dpi, mice were dissected, and the brain tissues were collected for assessment of antiviral activity. Results demonstrated that 10 mg/kg tetrandrine significantly suppressed HCoV‐OC43 replication in either RNA, protein or viral titer levels, while 5 mg/kg and 1 mg/kg tetrandrine showed less efficacy against HCoV‐OC43 with substantial inter‐individual variability among mice (Figure 8B–D). The positive control nirmatrelvir showed significant antiviral effects in all tested indexes as expected (Figure 8B–D).

Furthermore, we observed that HCoV‐OC43 infection induced a number of histological lesions in brain through hematoxylin and eosin (H&E) staining, especially neuron vacuolation (hydropic degeneration), perivascular cuffing, which commonly reported in neuropathological studies of COVID‐19 patients, and small hemorrhagic lesions. Nirmatrelvir and 10 mg/kg tetrandrine treatment significantly improved perivascular cuffing and neuron vacuolation, while administration of tetrandrine at 5 mg/kg or 1 mg/kg resulted in only limited neuroprotective effects (Figure 8E). Collectively, these findings suggest that a one‐time‐tetrandrine treatment can effectively inhibit HCoV‐OC43 in vivo, and it is comparable to that of nirmatrelvir.

## 3 Discussion

The viral pandemic has been a significant source of social, economic, and mortality stress for an extended period, underscoring the critical demand for effective therapeutic strategies. Among various approaches, drug repurposing has emerged as an efficient strategy in dealing with newly emerged virus outbreaks. In this study, we identified the natural compound tetrandrine as a potent broad‐spectrum inhibitor of coronaviruses. We verified that tetrandrine efficiently inhibited the infection of HCoV‐229E, HCoV‐OC43, and SARS‐CoV‐2, along with its major variants, across multiple cell lines (Figures 1, 2, 3). Furthermore, a single‐dose administration of tetrandrine in HCoV‐OC43‐infected neonatal mice showed significant antiviral effects in vivo (Figure 8). Importantly, the effective antiviral concentrations observed were substantially lower than the reported maximum tissue concentration (Cmax) of tetrandrine [29], thereby supporting its therapeutic potential against emerging coronavirus infections.

Mechanically, our data indicate that tetrandrine acted as an entry inhibitor by binding directly to the Spike protein and promoting its degradation (Figures 4 and 5). The drug‐resistance study confirmed this mechanism, and first reported two novel mutations, G688R and D814Y, within the S2 subunit of the Spike protein, which reduced the susceptibility of HCoV‐229E to tetrandrine while maintaining sensitivity to remdesivir and ribavirin (Figure 6). G688R was located within the fusion peptide (FP, residues Val686‐Asp711), affecting the function of host proteases [33, 34], while D814Y lied within the heptad repeat 1 (HR1, residues Phe769‐Asp870) region, which mediated the formation of the prefusion Spike trimer [33, 34]. In silico modeling suggested that tetrandrine forms binary or ternary complexes with S2 subunit residues (e.g., Arg689 and Asp814) and TMPRSS2 (Asp121), thereby interfering with Spike priming and subsequent membrane fusion. This is further supported by the observed disruption of Spike‐TMPRSS2 interaction upon tetrandrine treatment (Figure 7C–E). However, the mechanism by which G688R and D814Y mutations of tetrandrine‐resistant HCoV‐229E diminish tetrandrine efficacy may involve altered binding affinities among tetrandrine, Spike and TMPRSS2. While our docking and molecular dynamic simulations offer insights into this interaction, definitive structural validation using cryo‐EM or co‐crystallography is still required. Moreover, given the shared fusion mechanism among coronaviruses [35], it is plausible the additional, yet uncharacterized elements beyond the FP and HR1‐HR2 domains contribute to tetrandrine's inhibitory effect, meriting further investigation.

The in vivo efficacy of tetrandrine was further validated using a neonatal mouse model of HCoV‐OC43 infection (Figure 8). A single administration of tetrandrine at 10 mg/kg significantly reduced viral load in the brain and ameliorated characteristic neuropathological features such as neuronal vacuolation and perivascular cuffing, which mirror neurotropic manifestations observed in human coronavirus infections. This outcome is crucial in translating the in vitro antiviral potency of tetrandrine into a therapeutically relevant context. However, it is important to acknowledge that this model involves neonatal mice, and thus the pharmacokinetics, biodistribution, and potential toxicity of tetrandrine in adult animals or humans remain to be fully elucidated. Tetrandrine's limited aqueous solubility and known cardiotoxicity at higher concentrations may constrain its systemic application, highlighting the need for further formulation and safety optimization prior to clinical translation.

Tetrandrine has been previously characterized as a calcium channel blocker that inhibits two‐pore channels (TPCs), thereby interfering with the capsid disassembly and nuclear transport—processes essential for virus entry [27, 36]. Tetrandrine has been reported to robustly impede the cellular entry of various viruses, including Ebolavirus (EBOV), MERS‐CoV, polyomavirus, and SARS‐CoV‐2 [24, 25, 26]. In addition, it exhibited antiviral efficacy against African Swine Fever Virus (ASFV), primarily by suppressing macropinocytosis through inhibition of the PI3K/Akt signaling pathway [37]. These studies suggest that tetrandrine rely on different mechanisms to act against different viruses, and also remind us of the potential off‐target effects or toxicity of tetrandrine in human subjects require rigorous safety evaluation before clinical translation. Moreover, advancements in technology have enabled in silico evaluation to explore the potential functions of compounds and their selected targets in the viral lifecycle. Several studies have reported that tetrandrine exhibits a strong affinity for binding to viral components, including Spike glycoprotein, nucleocapsid protein, proteases 3CLpro, and helicase [38, 39]. Here, we proved that tetrandrine induced Spike to degradation; however, the detailed molecular mechanism by which tetrandrine induced Spike degradation remains to be elucidated in further work.

Additionally, our findings also partially exclude the possibility that tetrandrine's antiviral activity is a side effect of its phospholipidosis‐inducing property [28]. Phospholipidosis is known to disrupt lysosomal lipid metabolism and trafficking—the processes that indirectly impair the formation of double‐membrane vesicles essential for viral replication [40, 41], while in this paper we observed that entry was the main process affected by tetrandrine, and the in vivo efficacy in the HCoV‐OC43 mouse further supports the conclusion, as phospholipidosis inducible drugs are unlikely to be effective in vivo [40].

In summary, our study demonstrates that tetrandrine predominantly disrupts the conserved entry stage of the viral lifecycle by targeting Spike‐TMPRSS2‐mediated Spike priming and the subsequent membrane fusion. While the discovery of resistance‐associated mutations and strong in vivo antiviral activity reinforce its therapeutic potential, limitations remain—particularly regarding its pharmacokinetics, solubility, potential toxicity, and the need for further structural and mechanistic validation in other coronavirus species, including SARS‐CoV‐2. Nonetheless, this study not only provides valuable insights into viral entry mechanisms but also highlights the therapeutic promise of entry inhibitors in the broader context of coronavirus preparedness and drug development.

## 4 Materials and Methods

### 4.1 Cells

Human large cell lung carcinoma cell line H460 was generously provided by Professor Zhen Wang, while the hepatocellular carcinoma cell line Huh 7 and Huh 7.5 were obtained from Professor Zonggen Peng. Both researchers are affiliated with the Institute of Medicinal Biotechnology, Chinese Academy of Medical Sciences and Peking Union Medical College. The human ileocecal adenocarcinoma cell line HCT‐8 was acquired from National Collection of Authenticated Cell Cultures (Shanghai, China). The Vero E6 cell line, derived from the kidney of the African green monkey, was supplied by Institute of Medical Biology, Chinese Academy of Medical Science (Kunming, China). An HEK293T‐derived cell line stably expressing recombinant hACE2 (HEK293T‐hACE2) was purchased from Delivectory Bioscience Inc. (Beijing, China). All cell lines mentioned above were maintained in Dulbecco's modified Eagle medium (DMEM, Invitrogen, Carlsbad, CA, USA) or RPMI Medium 1640 basic (1×) (Thermo Fisher Scientific, Waltham, MA, USA), each supplemented with 10% fetal bovine serum (FBS) and antibiotics (100 U/mL penicillin and 100 mg/mL streptomycin) and incubated at 37°C in 5% CO_2_ incubator.

### 4.2 Viruses

The HCoV‐229E strain (VR740) was obtained from the American Type Culture Collection (ATCC). The HCoV‐OC43 strain (VR1558) was generously provided by Dr. Xuesen Zhao of Beijing Ditan Hospital, Capital Medical University (Beijing, China). The SARS‐CoV‐2 isolate (Genbank accession number: MT123290), along with its variant strains—B.1.1.7, B.1.351, and Omicron subvariants BA.5, EG.5, and XBB1.16—was preserved and utilized at the Institute of Medical Biology, Chinese Academy of Medical Science (Kunming, China). Pseudotyped SARS‐CoV‐2 and vesicular stomatitis virus (VSV) were acquired from Dilivectory Biosciences Inc. (Beijing, China).

### 4.3 Compounds and Plasmids

Tetrandrine, ammonium chloride (NH_4_Cl), and remdesivir were obtained from MedChemExpress (Monmouth Junction, NJ, USA). Ribavirin and CHX were sourced from Sigma‐Aldrich (St. Louis, MO, USA).

The plasmids pCMV3‐HCoV‐229E Spike‐Flag, pCMV3‐HCoV‐229E NP‐HA, pCMV3‐Flag‐APN, and pCMV‐3‐TMPRSS2‐HA were acquired from SinoBiological (Beijing, China). Additionally, the plasmids pLV‐myc‐HCoV‐229E Spike‐luci, pLV‐myc‐HCoV‐229E Spike^G688R^‐luci, pLV‐myc‐HCoV‐229E Spike^D814Y^‐luci, and pLV‐myc‐HCoV‐229E Spike^G688R/D814Y^‐luci were obtained from Inovogen Tech. Co. (Chongqing, China).

### 4.4 Cytotoxicity Test

The cytotoxicity of tetrandrine in the specified cell lines was detected by PreBlue kit Cell Viability Assay kit (Invitrogen, Carlsbad, CA, USA). Cells were seeded into 96‐well culture plates and allowed to adhere overnight under standard culture conditions. Following incubation, various concentrations of tetrandrine were applied to the cells and maintained for 48 h. Cell viability was then measured using the PreBlue assay, and the 50% cytotoxic concentrations (CC_50_) values were determined for each cell line.

### 4.5 Virus Infection

H460 and HCT‐8 cells were seeded at a density of 1.5 × 10⁵ cells/mL, while Huh7, Huh7.5, and Vero E6 cells were plated at 2 × 10⁵ cells/mL into appropriate culture plates and incubated overnight. HCoV‐OC43 was used to infect H460 and HCT‐8 cells, whereas Huh 7 and Huh 7.5 cells were exposed to HCoV‐229E. Vero E6 cells were infected with SARS‐CoV‐2, including its various mutant strains. Different multiplicities of infections (MOIs) were applied based on the virus type and experimental conditions to investigate the antiviral efficacy and underlying mechanisms of the compound under study.

### 4.6 qRT‐PCR

Total RNA was isolated using the RNeasy Mini Kit (QIAGEN, Hilden, Germany). The expressional levels of the target genes were analyzed by TransScript II Green One‐Step qRT‐PCR SuperMix or TransScript Taqman One‐Step qRT‐PCR SuperMix (TransGen Biotech, Beijing, China), as appropriate. Reactions were carried out on the ABI 7500 Fast Real‐time PCR system (Thermo Fisher Scientific). The specific primer sequences utilized are listed in Table S1.

### 4.7 Immunoblotting

To confirm the protein expression levels, cellular lysate was prepared using M‐PER Mammalian Protein Extraction Reagent (Thermo Fisher Scientific) supplemented with a 1× Halt protease Single‐Use inhibitor cocktail (Thermo Fisher Scientific). The antibodies employed for immunoblotting analysis included the following: anti‐glyceraldehyde‐3‐phosphate dehydrogenase (GAPDH) (Cell Signaling Technology, 1:1000), anti‐β‐actin (Cell Signaling Technology, 1:1000), anti‐HCoV‐229E nucleocapsid protein (NP, SinoBiological, Beijjing, China, 1:1000), anti‐HCoV‐OC43 nucleocapsid protein (NP, Millipore, Billerica, MA, USA, 1:1000), anti‐pan‐coronavirus Spike (S, SinoBiological, 1:1000), anti‐SARS‐CoV‐2 nucleocapsid protein (NP, SinoBiological, 1:1000), anti‐aminopeptidase N (APN/CD13, SinoBiological, 1:1000), and anti‐TMPRSS2 (Santa Cruz Biotechnology, Inc., Texas, USA, 1:1000). Immunoblotting procedures conducted according to previously established protocols [42].

### 4.8 Immunofluorescence Assay

H460, HCT‐8, Huh 7, and Huh 7.5 cell lines were cultured on glass coverslips (Thermo Fisher Scientific) and allowed to adhere overnight. The following day, cells were simultaneously infected with the designated virus and treated with the corresponding concentration of tetrandrine. After 24 h post‐infection, cells were rinsed and subsequently fixed. For immunofluorescence analysis, the following primary antibodies were utilized: anti‐HCoV‐229E nucleocapsid protein (NP, SinoBiological, 1:200), anti‐HCoV‐OC43 nucleocapsid protein (NP, Millipore, 1:200), and anti‐dsRNA (SCICONS, Szirák, Hungary, 1:200). Immunofluorescence staining was carried out as previously described [42], and the fluorescence images were acquired using an Olympus IX71 microscope (Olympus, Japan).

### 4.9 Time Course Assay and Time‐of‐Addition Assay

The stage(s) of viral replication targeted by tetrandrine were investigated through time‐of‐addition and time‐course assays, which assessed the impact of delayed tetrandrine treatment on viral NP expression (Figure 5A,D). In brief, H460 and Huh 7 cells were infected with HCoV‐OC43 and HCoV‐229E, respectively. In the time‐of‐addition assay, tetrandrine was administered either simultaneously with viral inoculation or at various time points following infection (Figure 5A). For the time‐course assay, cells were treated with tetrandrine for defined intervals only (Figure 5D). All samples were collected at 12 h post‐infection, and NP expression levels were analyzed via immunofluorescence staining.

### 4.10 Inducement of Drug‐Resistant Virus Strain

Huh 7 cells were seeded into 3.5‐cm culture dishes at a density of 2 × 10^5^ cells per dish and incubated overnight to allow for attachment. The following day, cells were infected with HCoV‐229E at a MOI of 0.5 and treated concurrently with 2 µM tetrandrine, a concentration previously determined to exhibit antiviral activity without cytotoxicity. After 48–72 h post infection (dpi), when extensive cytopathic effect (CPE) was observed, the culture supernatant (containing the virus) and the cells were collected together and stored at −80°C, which was designated as the first generation (passage 1) of tetrandrine‐treated virus. The collected virus from passage 1 was then thawed and used to infect a batch of Huh 7 cells prepared under identical conditions. In each round of infection, tetrandrine treatment was repeated, and its concentration was incrementally increased across passages to apply selective pressure. The concentration of tetrandrine was raised stepwise in accordance with the previously established cytotoxicity curve, until reached the maximum non‐toxic concentration and no longer inhibited viral replication. After multiple passages, when the virus could not be inhibited by the highest tolerable concentration of tetrandrine, the strain was deemed to have acquired resistance. The final passage of virus was collected, thawed, and used to infect a fresh Huh 7 cells without tetrandrine. At 24 dpi, the infected cells were harvested, and RNA was extracted, which was then subjected to sequencing and alignment (MAGORBIO, Shanghai, China).

### 4.11 Pseudovirus Infection and Luciferase Detection Assay

HEK293T cells stably expressing human ACE2 were seeded into 96‐well plates (white‐walled with clear bottoms) and subsequently infected with lentiviral particles pseudotyped with SARS‐CoV‐2 Spike protein or VSV‐G glycoprotein, in the presence or absence of tetrandrine. Ammonium chloride (NH_4_Cl) served as a positive control. At 24 h post‐infection, cells were lysed using 20 µg/well of lysis buffer (Promega, Madison, WI, USA) for 15 min. Following lysis, 50 µL/well of luciferase substrate (Promega) was added. Firefly luciferase activity was the quantified using a luminometer (EnSpire, PerkinElmer, Waltham, MA, USA).

### 4.12 Protein Degradation Detection

Huh 7 cells were transfected with the specified plasmids and incubated for 24 h. Cycloheximide (CHX) was then added in combination with either tetrandrine or DMSO, and cells were incubated for varying durations (0, 2, 4, 6, 8, 10, and 12 h). Following treatment, cells were harvested, and total proteins was extracted using M‐PER Mammalian Protein Extraction Reagent (Thermo Fisher Scientific) supplemented with 1× Halt Protease Inhibitor Single‐Use Cocktail (Thermo Fisher Scientific). Protein samples were subsequently subjected to immunoblotting analysis.

### 4.13 Immunoprecipitation

Huh 7 cells were transfected with the specified plasmids and incubated for 24 h. Afterward, cells were harvested and lysed on ice for 30 min using M‐PER Mammalian Protein Extraction Reagent (Thermo Fisher Scientific) supplemented with Halt Protease Inhibitor Single‐Use Cocktail (Thermo Fisher Scientific). Lysates were then centrifuged at 12,000 × *g* for 15–20 min, and the resulting supernatants were incubated overnight with 4 µg of the designed antibody. Magnetic beads were added to the mixture and incubated for 3 h, followed by washing with buffer to remove unbound material. Finally, the bead‐bound complexes were boiled for 10 min and subjected to immunoblotting analysis.

### 4.14 SPR

SPR analysis was conducted using the Reichert4 SPR system (Reichert, Buffalo, NY, USA). All reagents were prepared with ultrapure water produced by the Master Touch‐S15UVF purification system. The running buffer consisted of 1% DMSO in PBST (phosphate‐buffered saline, pH 7.6, containing 0.05% Tween‐20 and 1% DMSO), and was filtered through a 0.22‐µm membrane prior to use. Target proteins—including Spike proteins form SARS‐CoV‐2, HCoV‐229E, and HCoV‐OC43—were diluted in 200 µL of 0.25 µg/µL sodium acetate buffer (pH 4.5) and covalently immobilized onto the carboxymethyl dextran surface of a CM5 SPR chip via standard amine coupling chemistry, achieving an immobilization level of approximately 6000 response units (RU). Residual reactive sites on the sensor surface were blocked with ethanolamine. All measurements were carried out at a controlled temperature of 25 ± 1°C. Tetrandrine was diluted in the running buffer to create a series of concentrations (25, 12.5, 6.25, 3.125, 1.5625, and 0.7815 µM), and each concentration was sequentially injected into the SPR flow channels at a rate of 25 µL/min to monitor real‐time binding responses. Recombinant Spike protein of SARS‐CoV‐2, HCoV‐229E, and HCoV‐OC43 were obtained from SinoBiological.

### 4.15 Molecular Docking

The three‐dimensional structure of coronavirus Spike protein (PBD ID: 6U7H; https://www.rcsb.org/structure/6U7H) and human TMPRSS2 were obtained from the Protein Data Bank. The crystal structure was protonated and energy‐minimized using the “Quickprep” tool in the Molecular Operating Environment (MOE) software. Both Protein and ligand structures were prepared under the AMBER10: EHT force field. Molecular docking was performed using the MOE “Dock” module to explore interaction between the target proteins and candidate molecules. During docking, proteins were represented using a coarse‐grained model, and interaction modes were identified via fast Fourier transform‐based search. Subsequently, the coarse‐grained model and the side chains of the interacting residues were refined. Up to 1000 conformations were initially retained based on the London δ scoring function. These poses were further optimized using the Induced Fit protocol followed by energy minimization, and binding affinities were estimated using the GB/VI scoring function. The top 100 docking poses were preserved, and the most favorable conformation was selected as the final docking result.

### 4.16 Molecular Dynamics (MD) Simulation

MD simulations were conducted using GROMACS 2022. The General Amber Force field (GAFF) was assigned to the ligands, while the Amber99sb‐ILDN force field was utilized for the protein receptors. Each protein–ligand complex was placed in a cubic simulation box filled with TIP3P water molecules, maintaining a minimal distance of 1.2 nm between the complex and the box boundaries. The resulting box dimensions were 100 Å × 100 Å × 100 Å. To achieve charge neutrality, five negative (Cl^−^) ions were added. Van der Waals interactions were calculated using a cutoff radius of 1 nm, and long‐range electrostatics were evaluated using the particle mesh Ewald (PME) method with a gird spacing of 0.16 nm. Nonbonded interaction neighbor lists were updated every 10 integration steps. All hydrogens bonds were constrained using the LINCS algorithm, and the integration time step was set to 2 fs. Periodic boundary conditions were enforced in all three directions. Energy minimization was performed using the steepest descent algorithm, followed by two equilibration steps: 100 ps under constant volume and temperature (NVT), and another 100 ps under constant pressure and temperature (NPT). The system temperature was regulated at 300 K using a Berendsen thermostat, while pressure was maintained at 1 bar using the Parrinello−Rahman barostat. A 100‐ns production simulation was subsequently carried out. To evaluate the structural stability of the protein–ligand complexes, root mean square deviation (RMSD), root mean square fluctuation (RMSF), radius of gyration (Rg), and hydrogen bonding interactions were analyzed. In addition, the molecular mechanics Poisson–Boltzmann surface area (MM‐PBSA) method was employed to estimate the binding free energy between each protein and its corresponding ligand.

### 4.17 Animal Studies

Mouse infection studies were conducted under animal biosafety level 2 (ABSL‐2) conditions. All procedures involving animals were carried out in accordance with protocols approved by the Animal Care and Welfare Committee of the Institute of Medicinal Biotechnology, Chinese Academy of Medical Sciences and Peking Union Medical College. Pregnant BALB/c mice at 16 days of gestation were obtained from SiPeiFu Biotechnology Co., Ltd (Beijing, China). To access the antiviral efficacy of tetrandrine against HCoV‐OC43 in vivo, 7‐day‐old neonatal mice were administered intranasally with tetrandrine at doses of 10, 5, or 1 mg/kg. The compound was dissolved in Kolliphor HS 15 (Sigma‐Aldrich, St. Louis, MO, USA) and administered immediately following intranasal infection with HCoV‐OC43 (1000 TCID_50_). Mice in the vehicle control group received an equivalent volume of the solvent alone post‐infection. Nirmatrelvir was included as a positive control. Each experimental group consisted of 5 mice. At 5 days post‐infection, animals were euthanized for analysis. Brain tissues collected from each group were fixed in 10% neutral buffered formalin for histopathological evaluation using H&E staining. Additional brain samples were snap‐frozen and stored for downstream analyses, including qRT‐PCR, immunoblotting, and viral titration to access viral burden and replication.

### 4.18 Statistics Analysis

Statistical analyses were conducted using GraphPad Prism version 9.0 (GraphPad Software Inc., San Diego, CA, USA). Densitometric Quantification of immunoblotting signals was carried out with Image J (National Institute of Health (NIH), USA). Data are presented as the mean ± standard deviation (SD). Comparisons among groups were assayed by one/two‐way analysis of variance (ANOVA), followed by Holm‐Sidak's post hoc test. A *p*‐value less than 0.05 was considered statistically significant.

## Author Contributions

Yuhuan Li is the PI of the study and directed the study. Jiandong Jiang also directed the study. Kun Wang designed and conducted the experiments, and wrote the main manuscript. Huiqiang Wang, Shuo Wu, Ge Yang, Haiyan Yan Lijun Qiao, Xingqiong Li, and Mengyuan Wu conducted the experiments and analyzed the data. All authors have read and approved the final manuscript.

## Ethics Statement

The animal experiments were approved by the Animal Care & Welfare committee of the Institute of Medicinal Biotechnology, Chinese Academy of Medical Sciences and Peking Union Medical College for the Ethics of Animal Care and Treatment (No. IMB‐20230601‐D_11_‐01).

## Conflicts of Interest

The authors declare no conflicts of interest.

## Supporting information



Supporting Information

## Data Availability

Data supporting the findings in this study are available from the corresponding authors upon reasonable request.
