# Supplementary material for: Disruption of Spike Priming in Virus Entry: Tetrandrine as a Pan‐Coronavirus Inhibitor
Source: MedComm (2020). 2025 Aug 31;6(9):e70353. doi: 10.1002/mco2.70353 (PMC12399569; doi:10.1002/mco2.70353)
Supplement: Supplementary file 1 — Supporting Information [file MCO2-6-e70353-s001.docx]

**Disruption of Spike Priming in Virus Entry: Tetrandrine as a Pan-coronavirus inhibitor**

Kun Wang^1^, Huiqiang Wang^1^, Shuo Wu^1, 2^, Ge Yang^1^, Haiyan Yan^1^, Lijun Qiao^1^, Xingqiong Li^1^, Mengyuan Wu^1^, Jiandong Jiang^1, 2, **^, Yuhuan Li^1, 2, *^

^1^ CAMS Key Laboratory of Antiviral Drug Research, Beijing Key Laboratory of Technology and Application for Anti-Infective New Drugs Research and Development, NHC Key Laboratory of Biotechnology of Antibiotics, Institute of Medicinal Biotechnology, Chinese Academy of Medical Sciences and Peking Union Medical College, Beijing, China

^2^ State Key Laboratory of Bioactive Substances and Functions of Natural Medicines, Institute of Medicinal Biotechnology, Peking Union Medical College and Chinese Academy of Medical Sciences, Beijing 100050, China

* Corresponding author: Yuhuan Li, PhD. Tel: 86-10-63010984; Fax: 86-10-63017302; Email: yuhuanlibj@126.com (Yuhuan Li).

No 1, Tiantan Xili, Beijing 100050, P.R.China

**Corresponding author: Jiandong Jiang, PhD. Email: Jiang.jdong@163.com (Jiandong Jiang)

No 1, Tiantan Xili, Beijing 100050, P.R.China

**Supplementary information**


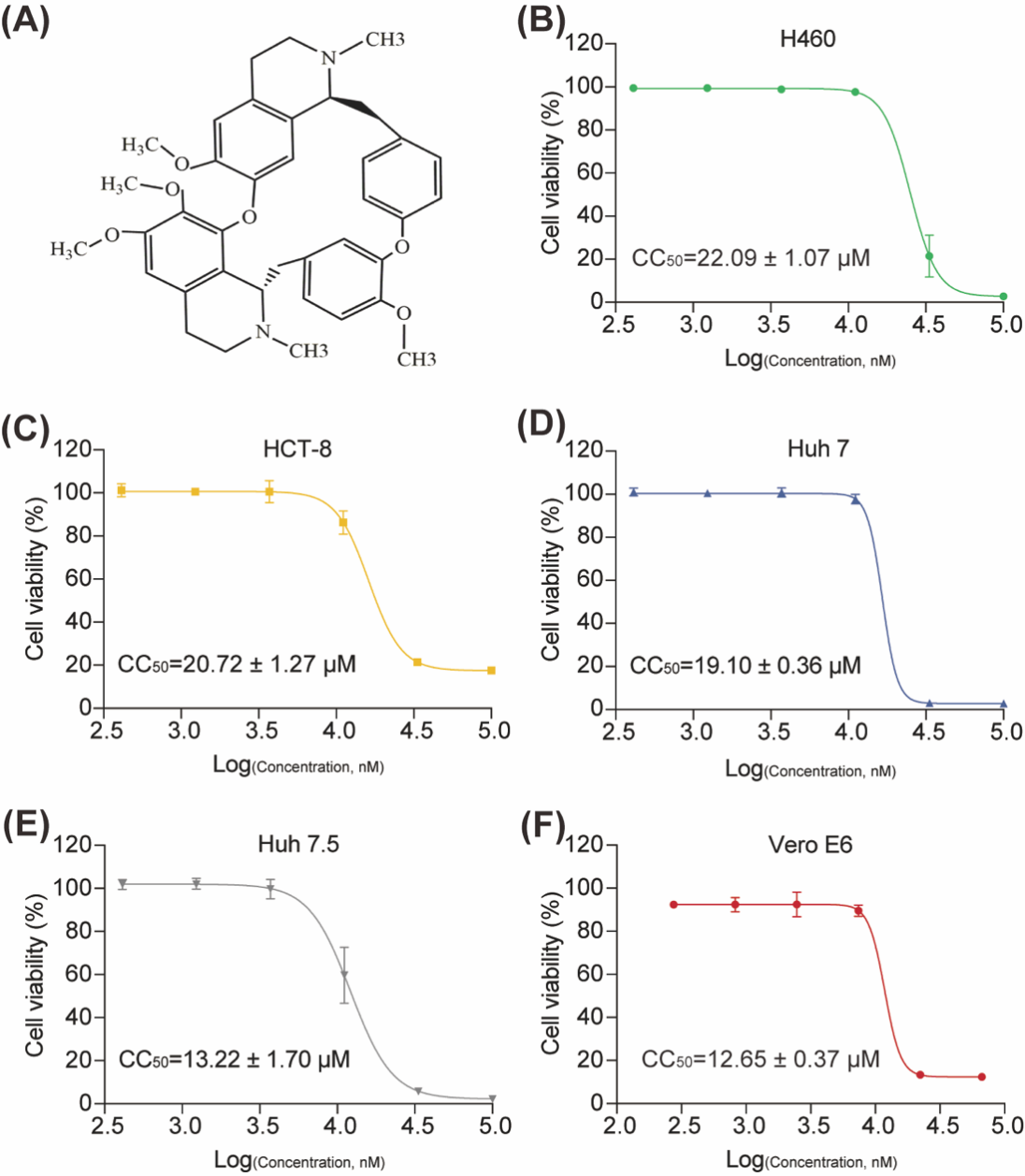


**Figure S1 Preliminary assessment for activity of tetrandrine against coronaviruses**

(A) The chemical structure of tetrandrine. The cytotoxicity (B-F) of tetrandrine on multiple cell lines was determined by preblue kit, the half-maximal cytotoxicity concentration (CC_50_) values were calculated by Reed and Muench methods. *n* = 3, the data represents the mean ± SD.

**Table S1 Primers used in qRT-PCR assay**

| Primer | Sequence |
| --- | --- |
| HCoV-229E *NP*-F | 5’-CGCAAGAATTCAGAACCAGAG-3’ |
| HCoV-229E *NP*-R | 5’-GGCAGTCAGGTTCTTCAACAA-3’ |
| HCoV-229E *Spike*-F | 5’-ACCTGTGTCTATTGTGTCGCTACC-3’ |
| HCoV-229E *Spike*-R | 5’-CTTGCCACCGCCACTCTGAG-3’ |
| HCoV-OC43 *NP*-F | 5’-CGATGAGGCTATTCCGACTAGGT-3’ |
| HCoV-OC43 *NP*-R | 5’-CCTTCCTGAGCCTTCAATATAGTAACC-3’ |
| HCoV-OC43 probe | 5’-TAMRA-TCCGCCTGGCACGGTACTCCCT-BHQ2-3’ |
| HCoV-OC43 *Spike*-F | 5’-TGTTGCCTCCACTGCTCTCAG-3’ |
| HCoV-OC43 *Spike*-R | 5’-ACCTGCTGCTGCTGTCCAAG-3’ |
| SARS-CoV-2 *NP*-F | 5’-GGGGAACTTCTCCTGCTAGAAT-3’ |
| SARS-CoV-2 *NP*-R | 5’-CAGACATTTTGCTCTCAAGCTG-3’ |
| SARS-CoV-2 probe | 5’-6-FAM-TTGCTGCTGCTTGACAGATT-TAMER-3’ |
| h*GAPDH*-1-F | 5’-CTCTGGAAAGCTGTGGCGTGATG-3’ |
| h*GAPDH*-1-R | 5’-ATGCCAGTGAGCTTCCCGTTCAG-3’ |
| h*GAPDH*-2-F | 5’-CGGAGTCAACGGATTTGGTCGTAT-3’ |
| h*GAPDH*-2-R | 5’-AGCCTTCTCCATGGTGGTGAAGAC-3’ |
| h*GAPDH*-2 probe | 5’-TAMRA-CCGTCAAGGCTGAGAACGG-BHQ2-3’ |
| murine *GAPDH*-F | 5’-CTCTGGAAAGCTGTGGCGTGATG-3’ |
| murine *GAPDH*-R | 5’-ATGCCAGTGAGCTTCCCGTTCAG-3’ |
| mk*GAPDH*-F | 5’-CTGTTGCTGTAGCCAAATTCGT-3’ |
| mk*GAPDH*-R | 5’-ACCCACTCCTCCACCTTTGAC-3’ |
